# Factors Affecting Dietary Practices in a Mississippi African American Community

**DOI:** 10.3390/ijerph14070718

**Published:** 2017-07-03

**Authors:** Monique White, Clifton Addison, Brenda W. Campbell Jenkins, Frances Henderson, Dorothy McGill, Marinelle Payton, Donna Antoine-LaVigne

**Affiliations:** 1 Center of Excellence in Minority Health and Health Disparities, School of Public Health, Jackson State University, 350 West Woodrow Wilson Drive, Suite, 2900B, Jackson, MS 39213, USA; clifton.addison@jsums.edu (C.A.); brenda.w.campbell@jsums.edu (B.W.C.J.); henfra@att.net (F.H.); Djceo@ibshealthy.com (D.M.); marinelle.payton@jsums.edu (M.P.); donna.antoine-lavigne@jsums.edu (D.A.-L.); 2 Jackson Heart Study Community Outreach Center, School of Public Health, Jackson State University, 350 West Woodrow Wilson Drive, Suite, 2900B, Jackson, MS 39213, USA; 3 Jackson Heart Study Graduate Training and Education Center, School of Public Health, Jackson State University, 350 West Woodrow Wilson Drive, Suite, 2900B, Jackson, MS 39213, USA

**Keywords:** public health, disparities, dietary practices, cardiovascular disease, African Americans

## Abstract

This study examined the practices, personal motivation, and barriers of African American communities in Mississippi regarding their dietary practices. We selected the Metro Jackson Area comprised of Hinds, Madison and Rankin Counties because it is a combination of urban and rural communities. The sample consisted of 70 participants from seven sites. A total of seven focus groups responded to six questions to assess practices, personal motivation, and barriers to dietary practices: (1) Where in your community can you access fresh fruits and vegetables? (2) How many meals a day should a person eat? (3) What would you consider to be a healthy breakfast, lunch and dinner? (4) What would you consider to be a healthy snack? (5) What do you consider to be your motivations for eating healthy? (6) What do you consider to be your barriers to eating healthy? Each of the seven focus groups consisted of 6 to 12 participants and provided details of their dietary practices. The focus group interviews were digitally-recorded. The recorded interviews were transcribed. The majority of the participants stated that there is a limited availability of fresh fruits/vegetables in rural areas because of a shortage of grocery stores. When they do find fruits, they are priced very high and are unaffordable. Even though health conditions dictate food frequency and portion size, community members feel that individuals should eat three good balanced meals per day with snacks, and they should adhere to small portion sizes. While the desire to attain overall good health and eliminate associative risks for heart disease (e.g., diabetes, obesity) are personal motivations, the cost of food, transportation, age, and time required for food preparation were seen as barriers to healthy eating. Decisions regarding meal choice and meal frequency can have an impact on long-term health outcomes. Health promotion programs should become an integral part of academic- community collaborative agreements.

## 1. Introduction

Cardiovascular disease (CVD) has caused a major public health burden that adversely affects finances, life expectancy, and healthcare, particularly in the case of African Americans [1]. Among adults aged 35–64, the highest rate of premature coronary heart disease occurred in African Americans living in the rural southern region of the United States [2]. The African American community in Mississippi is plagued with many challenges that affect health status. Among adults, obesity has become an epidemic worldwide and is closely related to dietary practices which also contribute to the development of diabetes, heart disease, hypertension, and cancer [3,4,5]. Childhood obesity has also become a major challenge to public health. Its high prevalence disproportionately affects low-income urban minority populations [6]. There is urgent need to increase community knowledge about risk factors for chronic diseases. Such an increase will draw attention to the importance of healthy eating, can lead to positive health benefits and the possibility of increasing lifespan. Research has shown that unhealthy diet is a primary risk factor for developing chronic disease that results in excessive morbidity and mortality among African Americans [7]. Other factors affect the dietary choices of African American low-income women who reside in urban communities [8]. Even though they desire to eat healthier and improve their health status, many African American women do not demonstrate healthy eating practices [7]. African American men also experience excess occurrence of preventable chronic diseases that can be attributed to poor health behaviors, like dietary practices. Poor dietary practices are associated with increased mortality risk [9]. Some people can achieve optimum health by eating a healthy diet that includes healthy servings of diverse fresh fruits and fresh vegetables [4]. 

Dietary patterns can be influenced by factors like accessibility, availability, affordability, and acceptability of healthy foods [10]. Dietary practices are also influenced by family cultural food preferences passed on from one generation to the next that are embedded in families and communities from generation to generation and place of residence [11]. Cultural and socio-economic factors like being poor are related to limited dietary choices and, subsequently lead to health disadvantages [12]. Most rural African American communities and individuals who are classified as poor generally have limited access to services or opportunities that would stimulate healthy practices [13]. Adults who live in rural areas experience a higher prevalence of obesity than adults who live in urban areas [14,15]. Many people who reside in poor African American communities lack facilities and resources that can enable them to adopt positive dietary practices because they live in poverty. In most of these communities, people have limited access to grocery stores and other outlets where fruits and vegetables are sold. So, in spite of the health promotion and education exposures, it is still difficult for many community members to adopt healthy dietary patterns [13,14,15]). The information obtained from the current study will provide insights into issues related to accessibility, availability, affordability, and acceptability of healthy foods in this African American community and facilitate interventions to help improve diet and ultimately decrease cardio-vascular disease.

Mississippi communities are well known for foods such as fried chicken, fried fish, cornbread, and pecan pie. A typical Mississippian meal generally includes these types of foods that are eaten regularly and in high quantities [16]. These types of negative dietary practices lead to a greater risk for cardiovascular disease, obesity, and the eventual development of metabolic syndrome [17]. In this study, we sought to examine the practices, personal motivation, and barriers of African American communities in Mississippi regarding their dietary practices. This is important because researchers believe that individuals can prevent heart disease by following a heart-healthy lifestyle [18]. In order to engage the community in health education, we first needed to explore their knowledge, perceptions, practices, beliefs, motivations, and barriers related to healthy eating.

## 2. Methods

### 2.1. Focus Group Participants and Selection Criteria

Data for this analysis were collected from 70 participants, the majority of whom were in age brackets from 50 to 75 from seven sites in Hinds, Madison, and Rankin County in Mississippi between May 2014 and February 2015. The Metro Jackson Area comprising Hinds, Madison and Rankin Counties was selected because it is a combination of urban and rural communities. Seven focus groups were asked to respond to six questions to assess dietary practices (see Table 1).

These focus groups consisted of six to twelve participants who were asked to comment on six questions. Recruitment focused on three locations in each of the three counties which totaled nine sites. The final sample consisted of participants from seven sites that comprised Innovative Behavioral Services (10), Ntense Fitness (8), Sweet Rest Baptist Church (13), Progressive Baptist Church (2 groups totaling 21), Canton Multi-Purpose Building (12) and Asbury United Methodist Church (6). These counties were selected due to their high rates of residents who engaged in poor dietary practices [19]. Poor dietary practices increase the risk for diseases, such as diabetes, cardiovascular disease and obesity [20], Hinds County is the largest county in the state; it is the location of the state capitol. Madison and Rankin Counties are rapidly growing rural and suburban counties [21]. Participants were recruited by networking with community partners, local churches, fitness centers and local government entities as well as through the dissemination of flyers and announcements at churches, and town hall events within each of the counties.

### 2.2. Focus Group Procedures

To elicit community members’ perceptions and knowledge regarding dietary practices, the program administrators developed a Community Questionnaire designed to collect demographic data such as: county of residence; gender; age; income; education; and employment. The focus group data collection spotlighted the community residents’ perceptions of dietary practices, beliefs, and awareness.

A qualitative study design was selected because it was best suited to enable our researchers to strive for an understanding of the whole [22] by engaging the community members to explore their own needs and aspirations [23] in a manner that acknowledges the community as a unit of identity, which a major principle of Community-Based Participatory Research [24]. Establishing the value of African American life can more effectively initiate discussions to address issues that improve the quality or quantity of that life [25]. These focus group interviews allowed us to hear multiple voices at one sitting [26], and participants got an opportunity to discuss issues and questions and build upon one another’s answers [27].

All participants were asked to complete and sign a consent form, thus abiding by the informed consent process. Participants were reminded that their participation was voluntary and they provided instructions for protecting confidentiality. The focus group interviews were digitally-recorded. The recorded interviews were transcribed by a professional transcriptionist.

### 2.3. Data Analysis

We analyzed the focus group data using Interpretive Phenomenology in order to arrive at interpretive descriptions of common practices and shared meanings that could reveal, enhance or extend our understanding of how participants perceived specific dietary practices and possibilities. We adapted Diekelmann’s seven stage process of data analysis [22]. Audio-recordings were transcribed verbatim into Microsoft Word (Microsoft Corporation, Albuquerque, NM, USA). Similarities and differences in perceived dietary practices, motivation, and barriers were identified within individual interview sites in the three counties (Asbury, Canton, IBS, Ntense, Progressive I, Progressive II and Sweet Rest), and then similarities and differences were examined between counties (Hinds, Madison, and Rankin Counties). A multi–disciplinary working group comprising six investigators from the Community Engagement Outreach Core of the Center of Excellence in Minority Health and Health Disparities Eliminating Health Disparities through Multi-Trans Disciplinary Approaches and a Qualitative Research Consultant (QRC) made up the writing group. This seven-person team participated in all aspects of data analysis and interpretation. A data file worksheet, by setting, for each of the three counties (Hinds, Madison and Rankin) was developed. Writing group members read through each county-specific focus group data file, and listed, on the worksheet, their interpretations for use in developing key themes for each focus group, according to the response to the questions. For this study, the data files were separated for interpretation according to the questions addressed in the focus group sessions (Table 1). Writing Group members reached consensus on their interpretations of the responses, and/or reconciled differences via discussion.

### 2.4. Ethics Statement

All community members gave their informed consent for inclusion before they participated in the study. The study was conducted in accordance with the Institutional Review Board at Jackson State University, Jackson, Mississippi, and the protocol was approved by the IRB (2014–2015).

## 3. Results

### 3.1. Focus Group Demographics

Focus group demographics are presented in Table 2. A total of 70 focus group members participated in interviews about dietary practices and possibilities for healthy eating. They were from the three counties that encompass the Jackson Heart Study: Hinds (N = 28); Madison (N = 27) and Rankin (N = 13). We did not have county data for two participants. Of the 70 participants, 56 were female, 14 were male; we are missing gender data for one participant. Forty-seven participants were 50 years of age or older, with 30 of them over the age of 60. In terms of income, we are missing data for eight participants; of the remaining 62, twenty percent earned less than $21,000 per year. Income for slightly more than half of the participants ranged from $21,000 to $39,000 per year. Participants with incomes of $40,000 to over $60,000 per year totaled 16 (26.0%).

Participants’ educational levels ranged from; some college, to Master’s degree, or higher. For most participants (78%), their educational level ranged from some college to Masters’ degree or higher, leaving 21% with a high school diploma, and one for whom we are missing data. While 30 of the 70 focus group participants were retired, 34 (49%) were employed, less than five percent were unemployed, and two were students. We are missing employment data for one participant.

All of the participants were from Hinds, Madison, or Rankin Counties, most were female aged 50 and older. The proportion of participants retired versus those who were employed was approximately a 50/50 split. Income for slightly more than half of the participants ranged from $21,000 to $39,000 per year.

### 3.2. Focus Group Responses

Focus group responses are reported according to six questions about dietary practices and healthy eating (see Table 1). The writing group and the QRC extracted common themes and patterns from the data file worksheets that described the essence of the experience for each county-specific focus group to the perceptions of dietary practices. In this section, we present the essence of the experience using exemplars and narrative descriptions that describe how the phenomenon was experienced by county-specific focus groups. We compared and contrasted the essence of the experiences between/among focus groups.

#### 3.2.1. Availability and Access of Fruits and Vegetables in the African American Community

The community members discussed, explored, and provided insight on their dietary practices and eating choices in their local communities. Members of the communities who participated in this focus group from the rural areas indicated that fresh fruits/vegetables are not readily available in the rural areas. All participants stated that they believe they can find fruits and vegetables within a 10-mile radius from their homes. According to one community member, “the community has fruits and vegetables, but if you try to purchase them from a side store, you would find that the prices are too high to buy”. Others believe that living in a rural, sparsely populated area/community may pose a challenge to obtaining fresh fruits and vegetables. “There is a lack of availability of fresh fruits/vegetables in rural areas because the rural areas do not have grocery stores”, intimated one community member. So, community members need to be taught “the alternatives if they can’t get fresh fruits; they should use canned fruits, but make sure that they rinse them”. As explained by one participant, those who live in rural areas have very limited choices when it comes to buying fresh fruits and vegetables. “When they do find them, fruits are priced high and that is turning people away from purchasing them”.

#### 3.2.2. Community Members’ Perceptions of Ideal Number of Meals a Day a Person Should Eat

Some participants suggested that community members should eat five small meals a day. However, the majority of the participants believed that three meals plus snacks should be consumed per day. They suggested that people with certain health conditions should pay particular attention to daily dietary requirements. The most frequent answer of the participants was that “the basic food group daily allowance is three good meals with snacks”, and individuals should eat “small portion sizes and balanced meals”. 

Some participants observed that certain health conditions should dictate food frequency and portion size. One community member displayed knowledge about the health dangers of an improper diet by mentioning that “diabetics should eat appropriately, but not too much because they can get ketoacidosis if they are not eating the correct food”. For people with certain chronic diseases, some of them recommended “four or five meals, but smaller portions; others recommended “six small meals”; Some community members believe that the number of meals/snacks should vary depending on chronic disease (diabetes, kidney disease, physical activity level, and the desire for weight loss; “Eating several meals a day is contingent on the desire to control weight”.

When asked what they considered to be the ideal meal per day, the community members appeared to be knowledgeable about what constitutes “healthy eating”. According to many participants, food choices were determined by food prices: The cost of food was considered by the participants as a determinant when choosing whether to eat healthfully/nutritiously or not. Participants provided their ideas of what constituted a breakfast, lunch, and dinner menu. The participants responded to open-ended questions about food choices without receiving any response choice/selection options. The food choices expressed were created by the participants themselves without prompting from the interviewer. For breakfast, the most common choices were: oatmeal, juice and tea/orange juice, eggs, grits, biscuit and fruit. The second most popular choices were: blueberries, walnuts, milk, half a bagel, half a banana, and a cup of coffee. The third most popular choices were: a vitamin shake, cereal, corn flakes and milk tea, green tea. For lunch, the most popular choices were: two vegetables/a sandwich/salad/turkey on wheat bread with lettuce and tomato and a diet soda/fruit. The second most popular choices were: baked chicken, fried chicken, grilled chicken, lima beans, greens, cornbread and some banana pudding. The third most popular choices were: a sandwich, ham, with wheat bread and some grape juice or orange juice, prune juice. For dinner, the top choices were: two vegetables and a meat/a salad. The second most popular choices were: turnip greens, green salad and chicken. The third most popular choices were: a Vitamin C drink like lemonade, grilled chicken and a salad.

#### 3.2.3. Community Members’ Suggestions for Breakfast, Lunch, and Dinner

The community provided their recommendations for the ideal breakfast. They feel that the ideal breakfast should include the following: oatmeal or eggs/fruits and protein—turkey sausage vegetables like spinach or tomatoes and maybe orange juice. Also included among the list of ideal breakfast foods were a cup of coffee and a piece of toast, as well as grits, biscuit, cereal, corn flakes, milk, and tea.

The community provided their recommendations for the ideal lunch. They feel that the ideal lunch should include the following: “Protein and vegetable/fuel foods, nuts, grains, lean meat, fruit, and chicken breast; fruit; wash it down with water of course”. Another recommendation for the ideal lunch was “sandwich/salad/turkey on wheat bread with lettuce and tomato and a diet soda/fruit”. Other ideal lunch suggestions included “baked chicken, fried chicken, grilled chicken, lima beans, greens, cornbread and some banana pudding, turkey, ham, grape juice or orange juice, prune juice”.

The community provided their recommendations for the ideal dinner. They feel that the ideal dinner should include the following: “Protein and vegetables/ and fruit, breads, cheeses and water, a carb, a cup of coffee, smoked sausage, toast with jelly, and orange juice. It should also include “a nice healthy protein shake”. Another recommendation is that the ideal dinner should include a meat, starch, a vegetable, a bread, and lemonade, or other “vitamin C drink”. There was also a recommendation that the ideal dinner should include vegetables, meat, salad, turnip greens, chicken, and water”.

#### 3.2.4. Perceptions of Healthy Snack in the African American Community

Most of the community members from the three counties, Hinds, Madison, and Rankin, provided responses that indicate that they have some familiarity with what constitutes a healthy snack. Community members agree that a mixture of fruits, juices, and nuts make up some of the healthy snacks that should be considered. They indicated that healthy snacks should include: “fruits (apple, strawberries, banana, blueberries), nuts, and vegetables”. In addition to the established healthy snacks components, community members also offered some unique recommendations that included “apple cider vinegar”, suggesting that home remedy beliefs should be an important consideration.

#### 3.2.5. Community Members Perception of Personal Motivations to Eating Healthy Individual and Family Health

The community members discussed several factors that provided motivation for them to seek modification of negative dietary practices to more positive ones. They provided the following as their personal motivations for seeking to improve their dietary practices: “desire to be more healthful (or helpful) for the body”; “to avoid diabetes”; “to lose weight for those with chronic diseases like high blood pressure and diabetes”; “need to have good lab results for primary care doctor’s visit”; “awareness of the struggles of members of the family with chronic diseases like diabetes”; “need to set an example for children to improve their lifestyles”; “motivated by parents who enjoyed great health throughout their lifetime”; “desire to avoid being overwhelmed by various physical disorders that would limit my independence, liberties”; “to get off medication; to lose weight; promote overall health and reduce CVD risk factors”. For most people, the desire to attain overall good health and eliminate associative risks for heart disease (diabetes, obesity) are personal motivations.

Community members suggested that their personal motivation for seeking good health through healthy eating might have been inspired by their desire to address health issues, particularly in the families where there is a family history of cardiovascular disease; for rural or urban individuals with limited education, such knowledge of family health history can be acquired by family members’ discussions to explore their medical history and the family’s history of cardiovascular disease. Their concern about overall health can be addressed and they would be in a better position to develop personal health goals. The family discussions can involve grandchildren, and other loved ones, and would provide information that can enable all of them to initiate practices for the prevention of risk factors for disease. They embrace the importance of pro-active health benefits, such as; “the desire to manage/prevent chronic disease to live a long life”; “to get off my medication/to lose weight/to maintain weight”.

#### 3.2.6. Community Members’ Perception of Barriers to Eating Healthy

Participants outlined their barriers to healthy eating. Some of them insisted that food is too costly and expensive to maintain the healthy diet that is recommended. They also believed that their lack of food choice control was a major barrier. Their addiction to good tasting food that may not be healthy limits their ability to eat healthy. The good tasting foods encourage people to indulge in double portions, illustrating their inability to accept limits on their food portion size. All of that is tied to the inability to change old family habits regarding food preparation and consumption. Even though some of them may know what to do, they hesitate to take action because they like to eat what tastes good. Some participants stated that they did not have the time to eat healthy meals. A barrier to eating healthy is the fact that preparing healthy meals takes time, and many people are too busy to find the time to prepare healthy foods. Another big problem resulted from the fact that many people were uninformed and lacked exposure to knowledge about the benefits and dangers of dietary practices. On the other hand, there were some who knew what to do because they were exposed to information, but still failed to make changes because it was inconvenient for them to make the necessary changes and because of the difficulty in changing old habits. In addition, those participants who resided in rural areas had limited access to groceries and healthy foods and had no transportation to get to areas that offered access to grocery stores and healthy foods. They would have to travel long distances, and just the thought of travelling long distances to purchase healthy foods limited their ability and willingness to seek and purchase healthy foods. 

## 4. Discussion

This study provided an opportunity to explore the perceptions of community members regarding dietary practices since dietary practices are considered a risk factor for the development of chronic diseases. In this project, the researchers treated the community as a unit of identity intending to build on the strengths and resources within the community. Community members were involved in a collaborative, equitable partnership with an empowering and power-sharing process that attempt to understand some of the social inequalities that existed. The process also sought to foster co-learning and capacity building among all the participants as they discussed the issues relating to their dietary practices. These findings illustrate some novel aspects and contributions to the field. There have been both cultural and socio-economic factors that impacted the community members’ ability to access and consume healthy meals. The community members from this tri-county area in Mississippi emphasized the importance of having adequate financial resources. In reference to accessing healthy foods they informed that there is a “lack of availability of fresh fruits/vegetables in rural areas”, and “if you purchase them from a side store the prices are too high to buy”. The discussions revealed that there are racial residential segregation and food deserts in the rural communities, where communities of color in particular lack access to stores selling healthy food. Living in a southern, rural community has some disadvantages, “a lot to do with location, with where people live”. As other community members indicated “a rural, sparsely populated area/community may pose a challenge to obtaining fresh fruits and vegetables”. Another community member indicated that “some people who live in rural areas have very limited choices that offer fresh fruits and vegetables, and fruits are priced high and that is turning people away from purchasing them”. One positive cultural advantage of living in a rural community is the fact that “the community in which the individuals reside have learned how to share in order to make it”.

Some cultural and generational influences on diet were apparent from the expressions and terms used in the discussion. The term “supper” was used by many of the participants in these communities instead of dinner, suggesting a cultural and generational preference that may also have different expectations and practices attached. Eating practices may be heavily influenced within local socio- structural contexts of the counties [28]. In examining the food choices people make, it has become clear that many people eat certain foods and prepare foods with certain ingredients based on acquired habit and family and community tradition. Many people do not consider selecting and consuming meals based on the health promotion qualities [29]. These dietary practices can be viewed as neighborhood-specific and identified with neighborhoods where only fast food and convenience stores are easily accessible. Because these foods are affordable, it is easier and cheaper for residents to buy and eat at these establishments. These communities have little choice because grocery stores selling fresh fruits and vegetable markets are not located in such communities. These individuals reside in communities that operate with limited choices relating to the types of foods available to them. So, the eating patterns of many communities are influenced by the personal, environmental, social, and cultural factors. Habits run in families and negative dietary practices, such as habitual eating of fast foods is often transferred from one generation to the next [30].

The participants made it clear that economics may play a role in the dietary decisions they make. Cost was considered by the participants as a determinant when choosing whether to eat healthfully/nutritiously or not and whether and how often they purchased fruits and vegetables, which is considered essential in ensuring a healthy diet. Their limited access to fresh fruits and vegetables expose deficiencies that conflict with the demands of some established programs to combat obesity. The Fresh Fruit and Vegetable Program is an important program supported by the Food and Nutrition Service (FNS) of the United States Department of Agriculture and state agencies designed to combat obesity. The Program highlights the importance of a variety of produce and complements the Institute of Medicine’s recommendations to provide healthier snack choices [15].

Residents from Hinds, Madison, and Rankin Counties believe that they live with severe disadvantages regarding the accessibility of healthy dietary choices, especially because they live in an area of the country that has the highest prevalence of obesity and cardiovascular disease. The chances of developing cardiovascular disease are reduced when community members make the choice to increase their daily intake of fruits and vegetables. Individuals who consumed eight or more servings of fruit and vegetables per day reduced their chances of suffering a heart attack or stroke by 30 percent in comparison to those who consumed less than 1.5 servings per day [31]. Green leafy vegetables and citrus fruits are recommended as the best selections to provide the most benefits. [31,32]. Studies on coronary heart disease and stroke concluded that consuming more than five servings of fruit and vegetables could reduce the chances of suffering coronary heart disease [32] and stroke [33] by 20 percent in relation to those who consumed less than three servings. The limited availability of fresh fruits and vegetables in this community limits some of the health benefits that good dietary practices can stimulate. There have been studies that support the consumption of a diet, fortified with fruits and vegetables that would provide certain health benefits to residents within the communities under examination in this study. The effect of a diet low in fat and high in fruits and vegetables on blood pressure was measured in the Dietary Approaches to Stop Hypertension (DASH) study [34]. Participants with elevated blood pressure who consumed the DASH diet were able to lower their systolic blood pressure by about 11 mmHg and their diastolic blood pressure by almost 6 mmHg. This adjustment in diet was as effective as taking medications. In the Optimal Macronutrient Intake Trial for Heart Health (OmniHeart), researchers found that diets that comprised of fruits and vegetables, as well as healthy unsaturated fat or protein had the effect of lowering blood pressure [35]. In this community that has a reputation for high prevalence of cardiovascular disease, increased availability of healthy foods is crucial to the implementation of prevention and intervention programs. 

Most participants were aware of the negative effects of poor dietary practices and the health effects that they produce. The recent increases in obesity are believed to be related to excessive consumption of high-energy dense foods [36,37]. They generally have the desire to attain optimum health and to reduce their risk for developing cardiovascular disease. To combat the prevalence of CVD and risk factors in the African American population in Mississippi, the community members agreed that it would be beneficial to explore the psychological and other self-regulatory features of the behaviors that can affect weight control. Some of these psychological features involve motivation and the accompanying desire to adopt new attitudes, set goals, while incorporating relevant behaviors [38].

Motivation allows the individual to self-regulate and self-determine eating behaviors and can be very effective as an intervention tool for addressing health disparities, risk factors for disease like obesity, and chronic disease like cardiovascular disease. Addressing the risk factors for disease requires sustained behaviors and changes in practices that can only be achieved through the use of motivation to enable lifestyle changes that are needed to reduce obesity, control blood pressure and diabetes.

The community members’ responses provided descriptions of common practices and shared meanings that could reveal, enhance and extend our understanding of their perceived dietary practices. Education on issues relating to dietary practices and the need to eliminate risk factors for chronic disease is important. However, good dietary practices also are contingent upon a positive social, economic, and psychological environment that allows the community members access to the healthy foods and the means to obtain them. In order to change negative dietary practices to more positive, heart healthy ones, community members will have to give up old habits, particularly regarding food preparation, in order to establish sound diet self-management [39]. Cultural factors are not fixed variables that occur independently from the contexts in which they are embedded [39]. The results of this study show that personal, structural, societal, and cultural factors influence food and dietary practices of communities and dictate everyday practices of this African American community. While these older community members struggle to protect their health, they face a number of barriers that affect their ability to take the steps necessary to improve and maintain their health. Research has reported about Americans living longer lives, but these older African American community members struggle to adopt the simple lifestyle changes that can help to ensure a healthier future. To ensure that the aging Mississippi community members are not neglected, community leaders and policy makers need to invest in public education, community-based programs, and policy interventions so that the aging population can be better served and can look forward to reaping maximum health benefits. 

### Limitations

The results of this study are qualitative, representing African American individuals from only three Mississippi counties, and should not be generalized to the larger Mississippi community in a statistical sense. One of the main limitations of the study is the age of the participants. The participants were over the age of 50, with many of them over the age of 60. This demographic has a number of implications both for dietary habits, generational effects, and prevention efforts, because according to some community members, advancing age limits one’s ability to participate fully in heart healthy activities. The results are useful for guiding interventions to address community perceptions and practices regarding nutrition and dietary practices and providing a foundation for future research relating to dietary practices.

## 5. Conclusions

This project allowed the researchers to involve the people of the community in a self-study of their own needs and aspirations regarding dietary practices. Some participants suggested that community members should eat five small meals a day. The majority of the participants stated that three meals plus snacks are adequate for daily consumption. It is understood that decisions regarding meal choice and meal frequency can have an impact on long-term health outcomes. Dietary experts believe that breakfast is a necessity because it activates the metabolism for the day and contributes to weight loss. Observational studies consistently show that people who skip breakfast are more likely to be obese than people who eat breakfast [31]. This is most likely due to the fact that those who skip breakfast are usually less health conscious and lack adequate knowledge about the negative impact of a poor diet. Many select snacks with little nutritious value like a doughnut at work and then have a big meal at a fast food restaurant at lunch. There is much debate and little certainty among the community members about the required frequency of meals that constitute healthy eating. It is important to note that multiple studies that have examined the benefits of eating many smaller meals compared to eating fewer larger meals have concluded that there is no significant effect on either metabolic rate or total amount of fat lost [32,33].

For these communities that are afflicted with chronic disease and have a high prevalence of cardiovascular disease, exercising self-management to modify negative behaviors is hinged upon motivation. Motivation is the single factor that can translate into behavior change and inspire success in intervention programs. In order to be successful to achieve fitness goals and to eliminate the risk factors that are preventable, individuals have to develop the motivation and the drive to make the individual commitment to make changes that are necessary for successful behavior modification to be realized. Human beings cannot be compelled by others to change negative behaviors into positive ones, such as what and how they eat, or what portion sizes they consume. Once community members become aware and are convinced of the negative impact of certain behaviors on health status, they can work towards developing the personal motivation to modify the identified negative behaviors to more positive ones. Recognizing and articulating the positive factors that can assist them to practice positive behaviors will assist in initiating and sustaining participation in prevention and intervention activities to eliminate health disparities and reduce the development of premature cardiovascular disease.

Dietary recommendations are a key element in the management of cardiovascular disease. Recent research has demonstrated that certain dietary patterns can influence cardiovascular health. It is possible to modify negative risk factors such as obesity, dyslipidemia, and hypertension, as well as factors involved in systemic inflammation, insulin sensitivity, oxidative stress, endothelial function, thrombosis, and cardiac rhythm [3,9]. While there is no single particular food that can decrease the risk of developing heart disease, some researchers have found that plant foods, especially wholegrain cereals, legumes, nuts, fruits and vegetables, may decrease the risk of heart disease. As obesity continues to increase in the United States, it is accompanied by numerous dietary trends responding to the rising prevalence [29]. Various dietary recommendations and selections have been reported in the literature and there have been major studies that investigated their effectiveness in modifying cardiovascular risk [30,40,41]. For this African American community, there is a limited availability of fresh fruits/vegetables in rural areas because their rural areas do not have grocery stores. When they do find fruits, they are priced very high and are therefore unaffordable. Even though health conditions dictate food frequency and portion size, community members feel that individuals should eat three good balanced meals per day with snacks, and they should adhere to small portion sizes. While the desire to attain overall good health and eliminate associative risks for heart disease (e.g., diabetes, obesity) are personal motivations, the cost of food, transportation, age, and time required for food preparation were seen as barriers to healthy eating.

### Implications for Practice and Future Research

According to some participants in a previous study, if healthy food tasted better, it would help them make the best possible food choices [8]. The participants in these Mississippi communities indicated that food cravings, taste, and appetite influenced their food choices. Limited knowledge about healthy foods and cravings for unhealthy foods, were barriers to making healthy dietary choices. The results of this study provide some ideas for researchers and public health professionals to consider for practice and future research. Health promotion programs should become an integral part of academic- community collaborative agreements. Understanding the stages of change can be helpful for developing interventions for improving eating habits [7]. Building awareness of the benefits and barriers of eating healthy is a good first step to address the importance of good dietary practices in maintaining cardiovascular health. Focusing on community intervention will help to address the widespread obesity and CVD epidemic in Mississippi and empower communities to build capacity to tackle the health disparity dilemmas that exist. These results will inform and shape an effective intervention program for improving dietary intakes [6]. Dissemination of information among community members about eating practices, and encouraging community members to be actively engaged in discussing and developing solutions within the local socio- structural contexts would also be an important step in addressing chronic disease risk reduction [28]. To monitor progress in approaches to eliminate health disparities, additional studies are needed to examine and promote healthy diets, nutrition, and weight management in African-American communities [42].

## Figures and Tables

**Table 1:** Focus group questions.

Focus Group Questions
Where in your community can you access fresh fruits and vegetables? How many meals a day should a person eat? What would you consider to be a healthy breakfast, lunch and dinner? What would you consider to be a healthy snack? What do you consider to be your motivations for eating healthy? What do you consider to be your barriers to eating healthy?

**Table 2:** Focus Group Characteristics.

Demographics Characteristics	Frequency	%
Counties		
Hinds	28	40.0
Madison	27	39.0
Rankin	13	19.0
Missing	2	2.0
Gender		
Female	56	80.0
Male	14	20.0
Age		
60 and above	30	43.0
50–59	40	57.0
Income		
Less than $21 K	12	17.0
$21,000–$39,000	34	49.0
$40,000–$60,000	16	23.0
Missing	8	1.0
Education		
Some College+	55	79.0
High School Grad	14	20.0
Missing	1	1.0
Employment Status		
Retired	30	43.0
Employed	34	49.0
Unemployed	3	4.0
Students	2	3.0
Missing	1	1.0
